# Insulin Pump Therapy Is Associated with Lower Rates of Retinopathy and Peripheral Nerve Abnormality

**DOI:** 10.1371/journal.pone.0153033

**Published:** 2016-04-06

**Authors:** Bedowra Zabeen, Maria E. Craig, Sohaib A. Virk, Alison Pryke, Albert K. F. Chan, Yoon Hi Cho, Paul Z. Benitez-Aguirre, Stephen Hing, Kim C. Donaghue

**Affiliations:** 1 Institute of Endocrinology and Diabetes, The Children’s Hospital at Westmead, Sydney, New South Wales, Australia; 2 Department of Paediatrics & Changing Diabetes in Children Program, Bangladesh Institute of Research and Rehabilitation in Diabetes, Endocrine and Metabolic Disorders, Dhaka, Bangladesh; 3 School of Women’s and Children’s Health, University of New South Wales, Sydney, New South Wales, Australia; 4 Discipline of Paediatrics and Child Health, University of Sydney, Sydney, New South Wales, Australia; 5 Ophthalmology Department, The Children’s Hospital at Westmead, Sydney, New South Wales, Australia; Monash University, Melbourne, Australia, AUSTRALIA

## Abstract

**Objective:**

To compare rates of microvascular complications in adolescents with type 1 diabetes treated with continuous subcutaneous insulin infusion (CSII) versus multiple daily injections (MDI).

**Research Design and Methods:**

Prospective cohort of 989 patients (aged 12–20 years; diabetes duration >5 years) treated with CSII or MDI for >12 months. Microvascular complications were assessed from 2000–14: early retinopathy (seven-field fundal photography), peripheral nerve function (thermal and vibration threshold testing), autonomic nerve abnormality (heart rate variability analysis of electrocardiogram recordings) and albuminuria (albumin creatinine ratio/timed overnight albumin excretion). Generalized estimating equations (GEE) were used to examine the relationship between treatment and complications rates, adjusting for socio-economic status (SES) and known risk factors including HbA_1_c and diabetes duration.

**Results:**

Comparing CSII with MDI: HbA1C was 8.6% [70mmol/mol] vs. 8.7% [72 mmol/mol]) (p = 0.7), retinopathy 17% vs. 22% (p = 0.06); microalbuminuria 1% vs. 4% (p = 0.07), peripheral nerve abnormality 27% vs. 33% (p = 0.108) and autonomic nerve abnormality 24% vs. 28% (p = 0.401). In multivariable GEE, CSII use was associated with lower rates of retinopathy (OR 0.66, 95% CI 0.45–0.95, p = 0.029) and peripheral nerve abnormality (OR 0.63, 95% CI 0.42–0.95, p = 0.026), but not albuminuria (OR 0.46, 95% CI 0.10–2.17, p = 0.33). SES was not associated with any of the complication outcomes.

**Conclusions:**

In adolescents, CSII use is associated with lower rates of retinopathy and peripheral nerve abnormality, suggesting an apparent benefit of CSII over MDI independent of glycemic control or SES.

## Introduction

Continuous subcutaneous insulin infusion (CSII) therapy has been used to treat diabetes since the late 1970s [1–3]. Over the last 15 years, CSII has increasingly been used in adults and children in an effort to optimise insulin delivery in type 1 diabetes. Several meta-analyses have demonstrated that CSII provides a slightly greater HbA_1_c reduction than multiple daily insulin injections (MDI) in adults [4–8] and a recent adolescent cohort study showed a sustained difference over 5 years [9].

The advantage of CSII is its ability to better mimic physiological insulin release, which may provide a more efficient supply of insulin to the tissues and minimize the risk of hypoglycemic events [4, 10]. The basal and bolus functions of the pump permit greater flexibility in timing and amounts of food intake and physical activity, allowing for greater variations in lifestyle [10]; and the data download function provides opportunity to review actual insulin delivery when counselling patients. A recent report from the Swedish National Diabetes Register shows a hazard reduction in pump users for cardiovascular mortality over a 6 year period without any difference in HbA1c at baseline [11]. In contrast to these advantages, adverse events have also been reported with insulin pump therapy including pump malfunction, infusion set/site failure and catheter infection [12, 13].

Over the same period that CSII use has increased, we have observed a reduction in some microvascular complications [14]. There is however no evidence demonstrating a long term effect of CSII on microvascular complications risk. Whilst some patients in the intensive treatment group of the Diabetes Control and Complications Trial (DCCT) used CSII, there was no benefit reported of CSII over MDI [15]. Thus, the objective of this study was to determine the impact of CSII on microvascular complications rates in adolescents with type 1 diabetes.

## Research Design and Methods

The study population consisted of adolescents with type 1 diabetes assessed for complications at The Children’s Hospital at Westmead from 2000 to 2014. Inclusion criteria were age between 12 and 20 years and diabetes duration of at least 5 years. This study was approved by the Ethics Committee of The Children’s Hospital at Westmead. Written informed consent was obtained from patients and their next of kin, caretakers or guardians on behalf of the minors enrolled in the study.

### Insulin therapy

Treatment was assigned as CSII or MDI (3 or more injections per day) if therapy had been instituted at least 12 months before complications assessment. For the purposes of describing the total population assessed for complications, a third category “other” was included, representing adolescents treated with MDI or CSII for less than 12 months or with 1–2 injections at the time of assessment.

### Complications assessment

Complications assessment was performed in patients during a 2 hour clinic visit, as described previously [14, 16]. Retinopathy was detected using stereoscopic fundal photography of seven fields; the IMAGEnet2000Lite system was used to digitalize images until 2011, and IMAGEnet R4 system thereafter. The photographs were graded by the same ophthalmologist according to the modified Airlie House classification of diabetic retinopathy [17].

Microalbuminuria was defined as mean albumin excretion rate (AER) ≥20 μg/min in at least two of the three timed overnight urine collections or albumin:creatinine ratio (ACR) ≥2.8mg/mmol (male) and ≥ 4.1 mg/mmol (female). Albumin was measured using the IMMAGE analyser (Beckman Coulter Australia) until 2003 and then using Immulite analyser (Siemens, Los Angles, CA, USA).

Peripheral nerve function was assessed by thermal threshold testing for hot and cold sensation at the left foot and vibration threshold testing at the left medial malleolus and left great toe using TSA—2001Neurosensory Analyzer Model TSA-II (Vibratory Sensory Analyzer—VSA-3000—Option). Because our nerve testing equipment changed in 2006, we have only included data collected after this time. Peripheral nerve abnormality was defined as sensory threshold scores above the 95^th^ percentile of non-diabetic adolescent controls tested previously in our laboratory [14].

Autonomic nerve function was assessed by measuring heart rate variability (HRV) using Lab Chart Pro 7 Analysis software (Ad Instruments, Sydney, NSW, Australia) from 2009 [18]. Participants underwent a 10-minute continuous ECG recording in supine position and measurements were taken in the morning at room temperature with patients breathing in a rested state in a quiet room. The entire 10-minute trace was used for analysis, with exclusion of ectopic beats (<500 ms, >1100 ms). Derived time domain measures were: standard deviation of mean NN intervals (SDNN) (where NN is determined from adjacent QRS complexes) and root mean squared difference of successive NN intervals (RMSSD), which are estimates of overall HRV. Frequency domain measures were: low frequency (LF), defined as >0.04 Hz and <0.15 Hz, and high-frequency (HF) components, defined as >0.15 Hz and <0.4 Hz, and the LF:HF ratio, considered to be an estimate of the relative sympathetic and parasympathetic balance. Autonomic nerve abnormality was defined as HRV scores below the 95^th^ percentile of non-diabetic adolescent controls [19].

Glycemic control was assessed by measurement of HbA1c using the Bio-Rad Diamat analyser (Bio-Rad, Hercules, CA). The nondiabetic range for HbA1c is 4–6% (20-42mmol/mol). Cholesterol was measured using a Beckman CX5 (1990–1999), using a Dimension RXL (2000–2005), and using a Vitros analyzer (Ortho Clinical Diagnostics) from 2005. High total cholesterol was defined as greater than 5.2 mmol/L.

Height and weight measurements were taken at each complication assessment, and body mass index (BMI) calculated as kilograms per squared meters. These measurements were converted to BMI z scores, using the 2000 Center for Disease Control (CDC) reference standards [20]. Systolic and diastolic blood pressure (SBP and DBP) z scores for age and sex were determined using the U.S. Task Force Report [21].

Socioeconomic status (SES) was classified using a postcode-based system according to the Australian Bureau of Statistics Socio-Economic Indexes for Areas (SEIFA) database [22]. The scale was used to classify participants socioeconomically disadvantaged group (low SES: deciles 1–3) and a socioeconomically advantaged group (high SES: deciles 4–10).

### Statistical analysis

Summary statistics are reported as mean ± standard deviation (SD) if normally distributed or median and interquartile range [IQR] for skewed data. Patients were stratified by treatment group (CSII, MDI, other) (Table 1). Comparisons across more than two groups were analyzed using ANOVA for normally distributed variables and the Kruskal-Wallis test for skewed data. Chi squared tests were used to compare categorical data across treatment groups.

**Table 1:** Characteristics and complication rates in adolescents with type 1 diabetes according to treatment.

	CSII (Min 12 months)	MDI (Min 12 months)	Other	P-value (MDI vs. CSII)	P-value (Across 3 Groups)
**Characteristics**					
Number	285	704	366	-	-
Male	128 (45)	332 (47)	178 (49)	0.521	0.640
Age (years)	16.6 [14.8–18.1]	17.1 [15.7–18.1]	16.4 [14.7–17.6]	0.007	< 0.001
Diabetes duration (years)	9.3 [7.3–12.1]	9.1 [7.1–12.1]	8.3 [6.7–11.1]	0.642	0.003
HbA_1c_ (%)	8.6 [7.9–9.7]	8.7 [7.8–9.6]	8.6 [7.8–9.7]	0.700	0.847
HbA_1_c (mmol/mol)	70 [63–83]	72 [62–81]	70 [62–83]	0.700	0.847
Insulin dose (units/kg/day)	0.80 [0.70–1.00]	1.10 [0.90–1.30]	1.10 [0.90–1.30]	< 0.001	< 0.001
Height SDS	0.25 ± 1.08	0.24 ± 1.01	0.09 ± 0.95	0.939	0.095
Weight SDS	0.80 [0.22–1.33]	0.85 [0.30–1.37]	0.72 [0.14–1.29]	0.621	0.085
BMI SDS	0.73 [0.04–1.28]	0.81 [0.29–1.29]	0.76 [0.18–1.23]	0.413	0.369
Cholesterol (mmol/L)	4.4 [3.9–5.0]	4.4 [3.8–5.1]	4.4 [3.8–5.0]	0.491	0.753
SBP SDS	-0.20 [-0.90–0.40]	-0.10 [-0.80–0.50]	0.00 [-0.80–0.70]	0.120	0.007
DBP SDS	-0.02 [-0.39–0.56]	0.34 [-0.18–1.29]	0.49 [-0.16–0.95]	< 0.001	< 0.001
SE disadvantage	40/284 (14)	139/704 (20)	61/361 (17)	0.037	0.095
**Complications**					
Retinopathy	46/273 (17)	151/678 (22)	81/350 (23)	0.062	0.116
Albuminuria	3/248 (1)	21/602 (4)	16/295 (5)	0.068	0.029
Autonomic nerve abnormality	55/229 (24)	57/207 (28)	24/73 (33)	0.401	0.311
Peripheral nerve abnormality	70/256 (27)	119/356 (33)	38/141 (27)	0.108	0.177

Data are n (%), mean ± SD, or median [IQR]. CSII, continuous subcutaneous insulin infusion; MDI, multiple daily injections.

Generalized estimating equations (GEE) were used to examine factors associated with the complication outcomes (retinopathy, microalbuminuria, peripheral nerve abnormality, and autonomic nerve abnormality) while taking into account multiple visits by individual patients. Explanatory variables included in the models were CSII vs. MDI, HbA1c, gender, age, duration of diabetes, height SDS, weight SDS, BMI SDS, SBP SDS, DBP SDS, insulin dose per kilogram body weight per day, and socioeconomic status (advantaged vs. disadvantaged).

## Results

A total of 1355 patients (53% female) were included in the study and results from 1520 complications assessments were included in the analysis. This represents 81% of eligible adolescents in our diabetes clinic.

Assessments were performed in 285 patients (21%) treated with CSII>12 months, 704 (52%) receiving MDI>12 months and 366 (27%) using other treatment (Table 1). There was no significant difference in HbA1c between patients treated with CSII vs. MDI (8.6% [70mmol/mol] vs. 8.7% [72 mmol/mol]; p = 0.70). Patients treated with CSII vs MDII were slightly younger (16.6 vs. 17.1 years; p = 0.007) and there was a higher proportion of socioeconomically advantaged patients (86% vs. 80%; p = 0.037) (Table 1).

**Fig 1:**
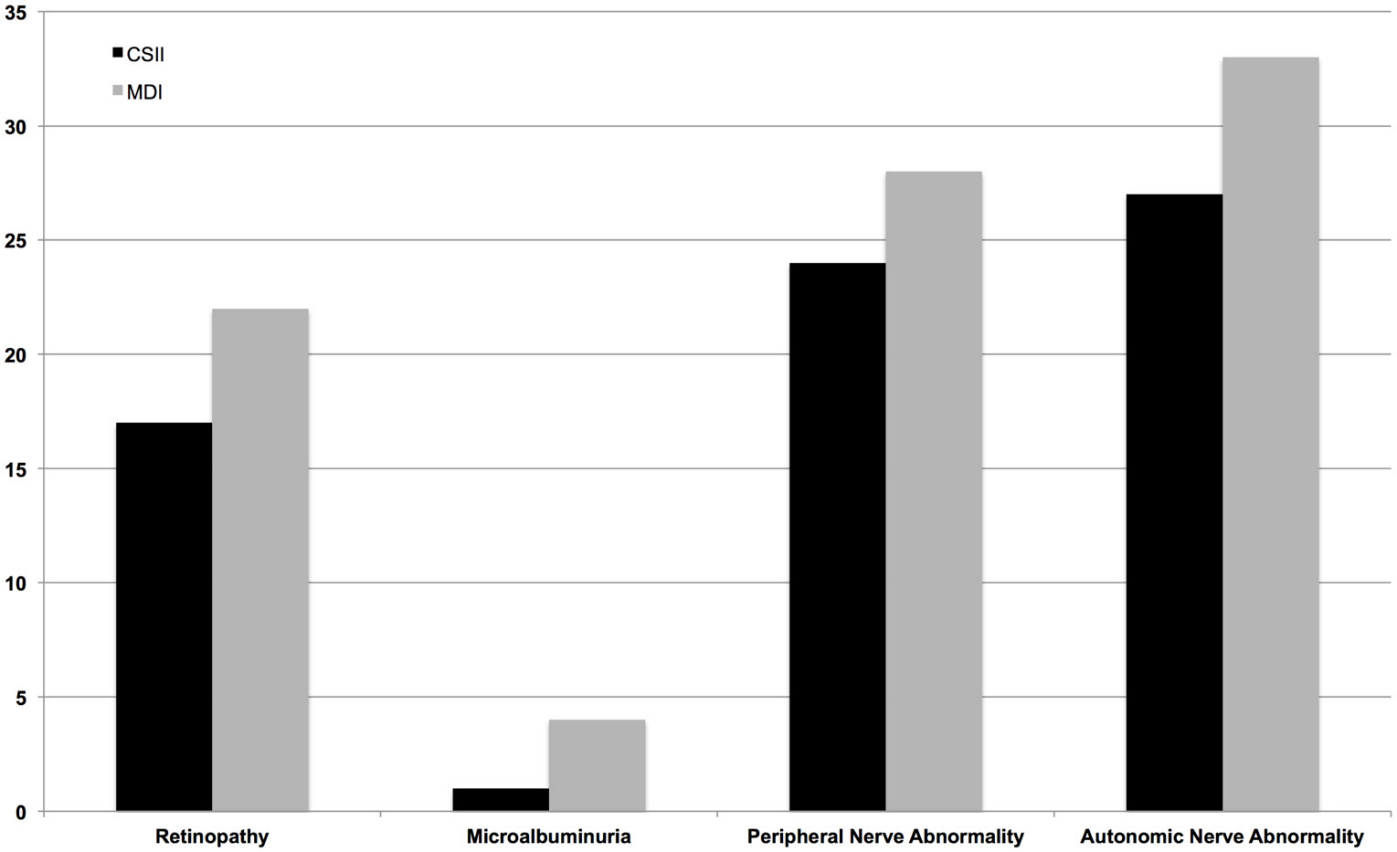
Rate of microvascular complications according to insulin delivery: CSII vs MDI.

The rate of microalbuminuria differed significantly across the three treatment groups, while rates of retinopathy, and peripheral and autonomic nerve abnormalities did not reach statistical significance. Comparing the intensive treatment groups, there was a trend towards a lower rate of retinopathy in those treated with CSII vs MDI group, (17% vs 22%, p = 0.062) and for microalbuminuria: (1% vs 4%, p = 0.068) (Fig 1).

### Multivariable analysis of complication outcomes

**Fig 2:**
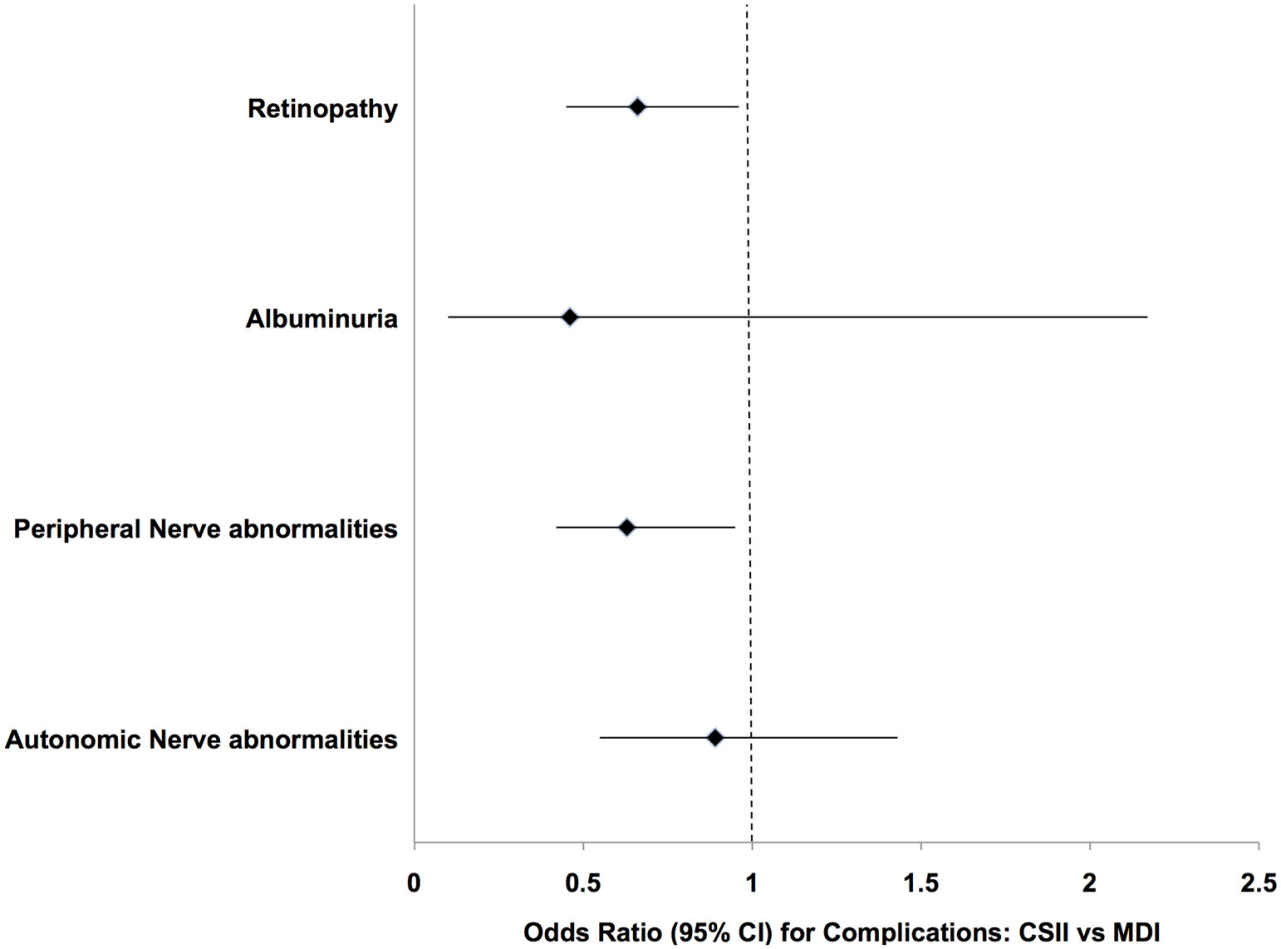
Effect of pump therapy compared to multiple daily injections for microvascular complications. Multivariable analysis (GEE), after inclusion of HbA1c and other confounders: significant reduction for retinopathy after allowing for HbA1c, age and duration; and significant reduction for peripheral nerve abnormalities after allowing for HbA1c, male gender and height SDS.

This analysis included 989 patients treated with either CSII or MDI for more than 12 months, with data on 1102 complications assessments (Table 2). The odds of retinopathy was lower in those treated with CSII vs MDI (OR 0.66; 95% CI, 0.45–0.95; p = 0.029), after inclusion of potential confounding variables in the model (higher HbA1c, older age and longer disease duration). The odds of peripheral nerve abnormality was lower in those treated with CSII (OR 0.63; 95% CI 0.42–0.95; p = 0.026), after inclusion of HbA1c, height SDS and male gender in the model. Neither autonomic nerve abnormality nor microalbuminuria were associated with CSII use in multivariable analysis (Fig 2). Autonomic nerve abnormality was associated with higher HbA1c, cholesterol, diastolic blood pressure SDS and female gender. Microalbuminuria was associated with higher HbA1c, insulin dose and diastolic blood pressure SDS.

**Table 2:** Generalized estimating equations for factors associated with microvascular complications in adolescents with type 1 diabetes.

Factor and outcome	OR (95% CI)	P value
**Retinopathy**		
Duration	1.13 (1.07–1.19)	< 0.001
Age	1.15 (1.05–1.27)	0.004
HbA_1c_	1.19 (1.08–1.31)	0.001
CSII vs. MDI	0.66 (0.45–0.96)	0.029
**Albuminuria**		
HbA_1c_	1.31 (1.01–1.70)	0.042
Insulin dose/kg/day	2.83 (1.29–6.22)	0.009
DBP SDS	1.88 (1.19–2.97)	0.007
CSII vs. MDI	0.46 (0.10–2.17)	0.330
**Peripheral Nerve Abnormality**		
HbA_1c_	1.11 (1.00–1.24)	0.060
Male	1.55 (1.07–2.24)	0.020
Height SDS	1.52 (1.26–1.82)	< 0.001
Insulin dose/kg/day	0.54 (0.29–1.01)	0.052
CSII vs. MDI	0.63 (0.42–0.95)	0.026
**Autonomic Nerve Abnormality**		
HbA_1c_	1.24 (1.08–1.44)	0.003
Male	0.52 (0.32–0.85)	0.008
DBP SDS	1.44 (1.07–1.95)	0.017
Cholesterol	1.37 (1.04–1.79)	0.025
CSII vs. MDI	0.89 (0.55–1.43)	0.627

CSII, continuous subcutaneous insulin infusion; MDI, multiple daily injections.

There was no association between socioeconomic advantage and any of the complication outcomes.

## Conclusions

In this longitudinal study of 989 adolescents with type 1 diabetes, CSII use for at least 12 months was associated with a significantly reduced risk of both retinopathy and peripheral nerve abnormalities, in addition to the effect of glycated haemoglobin. There was a trend for lower rates of other complications for those using CSII for more than 12 months. Importantly, socioeconomic status did not influence complications risk.

These findings, which imply a positive effect of CSII, were suggested in our previous report of microvascular complications in our clinic from 2005–2009 [14]. We reported lower rates of retinopathy and peripheral nerve abnormalities in those who were treated with CSII versus MDI, but this only reached significance in univariable analysis. Notably, fewer patients in our previous report were treated with CSII and treatment classification was not defined as a minimum of 12 months, but only by the therapy used at the time of complication assessment. Our current findings therefore provide more robust evidence for an association between CSII and lower complication rates.

Over the 15 year period of the study, the use of CSII has increased to 45% of our clinic population. Contrary to our expectation of a better glycated haemoglobin in the CSII users, their mean HbA1c was similar to the MDI users and patients receiving 1–2 daily injections. This supports the notion that CSII may protect from complications independent of its relatively modest effect on HbA1c reported by others [4–9]. We speculate that CSII is likely to confer a more stable glycaemic profile independent of glycated haemoglobin [23, 24].

Although HbA1C is currently the gold standard for assessing glycemic control, it does not provide a measure of the magnitude or frequency of fluctuations in blood glucose throughout the day. A relationship has been proposed between microvascular complications and postprandial glycemic spikes [23, 25], and this relationship may be undetected if only HbA1C values are assessed. In the DCCT, intensively treated patients had a significantly lower risk of retinopathy progression compared with the conventionally treated group for the same updated mean of HbA1C [26], suggesting that glucose variability may have contributed to the difference. This is consistent with our findings of reduced retinopathy prevalence in patients using CSII vs. MDI, despite the same HbA1C.

A lower rate of peripheral nerve abnormalities was found in the CSII users in multivariable analysis. This is in agreement with one previous study of individuals with type 1 diabetes, in which the onset and progression of micro- and macro-vascular complications were evaluated over an 11 year period. Glucose variability, calculated from 70 self-monitored measurements and standard deviation (SD) of blood glucose levels, was predictive of peripheral neuropathy but not nephropathy [27]. Their data suggested that glucose variability may be more detrimental to peripheral nerves than the glomerulus. Similarly, we did not demonstrate a beneficial effect of CSII on albuminuria, but this was also less common.

The proportion of socioeconomically advantaged patients was higher in CSII (86%) compared to MDI (80%); this was expected since CSII is predominantly used by patients in Australia who have private health insurance and are from higher socioeconomic groups. Importantly, socioeconomic advantage was not significant in any of the models for complications outcomes, suggesting this was not a confounder in the relationship between treatment modality and complications rates.

The strengths of this study include the large sample size and study design, with the cohort followed prospectively over 15 years from diagnosis. All patients were assessed at one center and comprehensive data collected at each visit. The study cohort is population based for Western Sydney Retinopathy assessment methods and assessment personnel were consistent throughout the study period. Peripheral nerve testing changed in 2006 so that different testing equipment was used in our earlier report to the equipment used in the current report [14]. The findings nevertheless are remarkably similar, with CSII compared to MDI therapy associated with 37% lower odds of peripheral neuropathy in both our previous [14] and current reports. Ideally, adolescents would have been randomised to a treatment group to determine the treatment effect on complications outcomes.

In conclusion, we have shown that CSII (insulin pump) use is associated with lower rates of early microvascular complications in a clinic population with type 1 diabetes. We hypothesize that reduced glycaemic variability in CSII users contributed to this reduction of complications, as there were no significant improvements in HbA1c. Longitudinal studies on CSII and MDI users, with inclusion of continuous glucose monitoring data, will provide further information on the benefits of CSII in this technological era. Future measures of glycaemic variability, such as the standard deviation of HbA1c, may further refine risk stratification. This is the first study demonstrating a specific benefit of CSII over MDI for microvascular complications in type 1 diabetes.

### Transparency Declaration

The corresponding author affirms that the manuscript is an honest, accurate, and transparent account of the study being reported; that no important aspects of the study have been omitted; and that any discrepancies from the study as planned have been explained.

### Licensing Statement

The Corresponding Author has the right to grant on behalf of all authors and does grant on behalf of all authors, a worldwide licence to the Publishers and its licensees in perpetuity, in all forms, formats and media (whether known now or created in the future), to i) publish, reproduce, distribute, display and store the Contribution, ii) translate the Contribution into other languages, create adaptations, reprints, include within collections and create summaries, extracts and/or, abstracts of the Contribution and convert or allow conversion into any format including without limitation audio, iii) create any other derivative work(s) based in whole or part on the on the Contribution, iv) to exploit all subsidiary rights to exploit all subsidiary rights that currently exist or as may exist in the future in the Contribution, v) the inclusion of electronic links from the Contribution to third party material where-ever it may be located; and, vi) licence any third party to do any or all of the above.
